# A retrospective study of sinonasal tumors in 182 dogs treated with stereotactic radiotherapy (3 × 10 Gy) (2010‐2015)

**DOI:** 10.1111/jvim.16838

**Published:** 2023-09-08

**Authors:** Hiroto Yoshikawa, Mary H. Lafferty, Lynn R. Griffin, Susan M. LaRue

**Affiliations:** ^1^ Department of Environmental and Radiological Health Sciences Colorado State University Fort Collins Colorado USA; ^2^ Flint Animal Cancer Center Colorado State University Fort Collins Colorado USA

**Keywords:** clinical signs of nervous system, cribriform plate lysis, nasal tumors, stereotactic radiotherapy

## Abstract

**Background:**

Stereotactic radiotherapy (SRT) is an emerging treatment for sinonasal tumors in dogs. Reported results regarding tumor control and incidence of acute and late radiation morbidities are inconsistent.

**Objectives:**

To determine treatment efficacy and prognostic indicators of SRT in dogs with sinonasal tumors and to quantify acute and late radiation morbidities.

**Animals:**

One hundred and eighty‐two client‐owned dogs with sinonasal tumors diagnosed cytologically, histologically, or radiographically that underwent SRT.

**Methods:**

Single‐arm retrospective study by reviewing medical records of dogs treated with SRT (10 Gy × 3) between 2010 and 2015. Kaplan‐Meier analysis was used to determine overall survival (OST; from the first day of SRT to death by any cause) and disease‐specific survival times (DSST; OST but censoring tumor/treatment‐unrelated death). Tumors were staged using modified Adams criteria.

**Results:**

Median OST and DSST of dogs treated with 1 course of SRT was 441 (95% CI: 389‐493 days) and 482 (428‐536 days) days, respectively with skin/oral cavity acute morbidities observed in 3% of dogs. DSST in dogs with stage 4 disease showed no statistical difference compared to other stages (*P* = .64). Oro‐nasal (n = 2) or naso‐cutaneous (n = 11) fistula development occurred in 7.1% of dogs with median time of 425 days (range: 83‐1733 days). Possible chronic rhinitis after SRT was recorded in 54 of 88 dogs (61%) where information was available.

**Conclusions and Clinical Importance:**

Results are comparable to other reports of treatment of SRT. Acute morbidities were minimal. Modified Adams stage scheme appeared to be inappropriate for prognostication for dogs with sinonasal tumors treated with SRT.

**Abbreviations**
CBCT: cone beam computed tomography
CI: confidence interval
CT: computed tomography
CTV: clinical target volume
DSST: disease‐specific survival time
GTV: gross tumor volume
IMRT: intensity modulated radiotherapy
KV: kilo‐voltage
LN: lymph node
MRI: magnetic resonance images
MV: mega‐voltage
OAR: organs at risk
OST: overall (any cause of death) survival time
PTV: planned target volume
SIB: simultaneously integrated boost
SRS: stereotactic radiosurgery
SRT: stereotactic radiotherapy
STs: survival times, including OST and DSST

## 1 INTRODUCTION

Sinonasal tumors in dogs are histologically diverse and predominantly tumors of epithelial and mesenchymal origin. These tumors can be expansive and could extend from the rostral nasal cavity into the frontal sinuses.^1^, ^2^ They can also invade the bones surrounding the nasal cavity, including cribriform plate with extension into the brain. Fractionated megavoltage (MV) radiotherapy using 3‐dimensional conformal treatment planning (3D‐CRT) has long been used for definitive treatment for sinonasal tumors in dogs despite severe acute radiation adverse events.^3^, ^4^ Since the development of intensity modulated radiotherapy (IMRT), studies using fractionated IMRT reported fewer acute adverse events and improved survival over historic reports.^4^, ^5^, ^6^ Stereotactic radiotherapy (SRT) is a more recent advanced technology. SRT is highly hypofractionated and requires a defined tumor target. The planning system must provide a rapid dose drop‐off from the tumor to the surrounding organs at risk (OARs). SRT also requires a method of tumor/target location verification at the time of treatment (stereotaxis).^7^ Unlike fractionated radiotherapy, which is delivered in 10‐20 daily fractions and uses fractionation to spare late‐responding normal tissues, SRT is delivered in 1‐5 fractions and spares normal tissues via avoidance.^8^ SRT is used in dogs and cats for a treatment of variety of tumors including sinonasal tumors.^9^, ^10^, ^11^, ^12^, ^13^, ^14^, ^15^, ^16^, ^17^, ^18^, ^19^ While the biology of SRT is still elusive, high dose/fraction irradiation can cause additional cell death through indirect methods, including vascular apoptosis and radiation‐induced tumor‐specific immunity.^20^, ^21^, ^22^, ^23^ The median ST of dogs with nonlymphomatous sinonasal tumors ranges from 240 to 399 days in 2 previous reports but^14^, ^19^ there is a high incidence of radiation adverse events. SRT improved quality of life and prolonged OST in 129 dogs of all life stages.^24^ The objective of this retrospective study was to provide additional data to inform optimization of future SRT protocols by evaluating treatment efficacy and documenting acute and late radiation adverse events.

## 2 MATERIALS AND METHODS

This is a single‐arm retrospective study performed at the Colorado State University Flint Animal Cancer Center. Medical records from databases and information from treatment plans were reviewed. Search criterion included dogs with a cytologically−/histopathologically‐confirmed sinonasal nonlymphomatous malignant tumors or those with computed tomography (CT) findings consistent with sinonasal tumors. CT criteria included contrast soft tissue attenuating masses and lysis of the turbinates and other bony structures. Dogs with distant metastases and/or tumor extending into regional lymph nodes were accepted. All dogs received a SRT prescription of 10 Gy × 3 daily and underwent treatment between January 2010 and August 2015. Preanesthetic evaluation included complete blood count, serum chemistry profile, and thoracic radiographs with modification depending on attending clinician. Dogs that had undergone multiple courses of SRT were analyzed separately. All dogs underwent CT scan for diagnostic purposes and SRT planning using a previously reported immobilization system.^25^ Regional lymph nodes (LNs) were cytologically assessed if LN were enlarged, firm, or displayed contrast heterogeneity. Additionally, normal nodes were aspirated and evaluated based on attending clinician's judgment. Dogs, if available for follow‐up, were evaluated for acute adverse events using the VRTOG acute events scoring system.^26^

**FIGURE 1:**
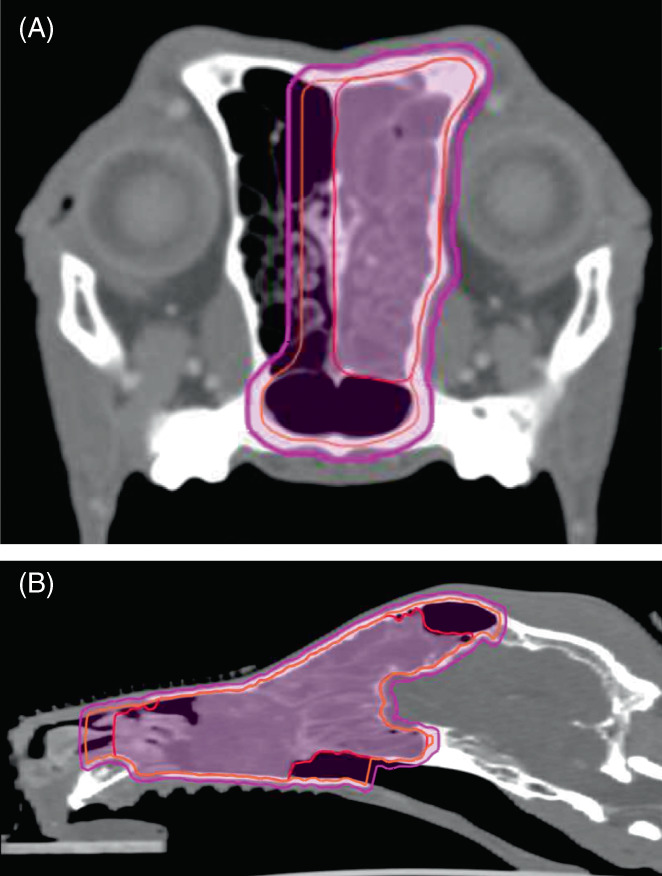
Representative images of tumor target contouring in (A) transverse and (B) sagittal planes. In this dog, clinical target volume was used. Red: gross tumor volume, Orange: clinical target volume, Magenta: planned target volume.

A series of transverse pre‐ and postcontrast simulation CT scan images with 2 mm slice thickness with no pitch was imported to a radiation planning workstation (Varian Eclipse ver. 11, Varian Medical Systems, Inc, Palo Alto, CA). Contoured OARs included skin (2 mm internal expansion from body surface, including palate mucosa), palate mucosa (2 mm thickness, including hard and soft palates), eyes, lenses, bones, brain, spinal cord, optic chiasm/nerve, and ear/cochlea. Mandibular and retropharyngeal LNs were contoured if treated simultaneously. Gross tumor volume (GTV) was defined as abnormal structure identified on the CT images including masses, contrasting enhancing areas, fluid in frontal sinuses, and regions of bony lysis. In some large tumors a simultaneous integrated boost (SIB) was applied. SIB is an area within the GTV where a higher dose than prescription is delivered. Criteria for SIB included a tumor with volume large enough to create at least a 1cm^3^ structure that did not include normal bone. However, because of the retrospective nature of the current study and attending clinician's judgment, not all dogs with a sinonasal tumor that is large enough for SIB creation received the boost. For dogs with possible intracranial invasion, multiple sequences of magnetic resonance images (MRI) were performed per attending clinician's judgment and coregistered with the simulation CT images to assist disease delineation in the intracranial space. No clinical target volume (CTV) expansion was applied until August 2011. CTV expansions were then applied because of our subjective impression of early local tumor recurrences because of possible geographic misses.^13^ The typical CTV expansion included 5‐10 mm of contralateral side of nasal cavity in dogs with unilateral tumor. The CTV expansion also included up to 1 cm expansion cranio‐caudally beyond the GTV; however, it did not extend into the cranial vault. Planning target volume (PTV) was created by applying 2 mm isotropic expansion to the CTV, limited within the body contouring. The GTV, CTV, and PTV were withdrawn 2 mm from the skin, mucosa, eyes, and brain. Planning OARs for spinal cord, optic chiasm/nerve, and ear/cochlea were created by applying 2 mm symmetrical expansion to the corresponding OARs. Constraints for OAR were obtained from AAPM Task Force 101 which was published in 2010.^7^ Before that we had a working copy of the aforementioned document (*Personal correspondence Brian Kavanagh*). Representative images of GTV, CTV, and PTV contouring is shown in Figure 1. Radiotherapy plans were generated through inverse planning technique with anisotropic analytical algorithm, static beams, and total 120 multileaf collimators with sliding leaf technique. Noncoplanar beams were used only when they showed a dosimetric benefit. Dose delivery accuracy was verified for each field with electronic portal imager and dedicated software (Aria portal dosimetry, Varian Medical Systems, Inc, Palo Alto, CA) with gamma tolerance <3%. All plans followed the recommendations of AAPM Task group 101, 142, and 147.^7^, ^27^, ^28^ All SRT plans were approved by an attending American College of Veterinary Radiology‐boarded veterinary radiation oncologist. Plan normalization was limited to less than 3% based on recommendations by in‐house medical physicist.

The SRT plans were delivered using a linear accelerator (Varian Trilogy, Varian Medical Systems, Inc, Palo Alto, CA) equipped with kilo‐voltage (KV) on‐board‐imaging devices. At each treatment session, after general anesthesia induction and setting the dog up on the treatment couch, dog positioning was verified before radiation dose delivery by image guidance with orthogonal kV‐kV images or cone‐beam CT (CBCT) images, based on attending clinician's judgment. After treatment delivery, dogs recovered from anesthesia and were monitored until discharged. Most dogs returned to their primary care veterinarians for follow up care. No standardized follow up schedule was followed.

Follow up information was collected by reviewing medical records and SRT plans. Primary care veterinarians, pet owners, or both of them were contacted to obtain additional information. Collected information was recorded and tabulated with a commercially available software (Office Excel, Microsoft, Redmond, WA). Collected information included signalment, clinical signs, histopathological diagnosis, staging test results, acute or late adverse radiation events, adjuvant therapy, and outcome. Possible prognostic indicators evaluated were age, body weight (BW), and values of blood work (each variable of CBC and chemistry) at the time of SRT, sex, disease laterality (unilateral vs bilateral), subcutaneous/submucosal space involvement (Y vs N), disease presence in the nasopharynx (Y vs N), presence of cribriform plate lysis (Y vs N), use of CTV (Y vs N), LNs simultaneously irradiated (Y vs N), presence of clinical signs of nervous system before SRT (Y vs N), and presence of clinical signs of nervous system after SRT (Y vs N). Modified Adams stage^29^ was further evaluated based on intracranial extension of the tumor with 4a including cribriform plate lysis and 4b including intracranial extension.^30^ The following dosimetric information was collected from the treatment planning software and also assessed for statistical significance as a predictive variable; volume of GTV and PTV (cc), tumor target volumes normalized to dog body weight (GTV/BW and PTV/BW) (cc/kg), calculated radiation doses to GTV and PTV (minimum; *D*_min_, 98%; *D*_98%_, maximum; *D*_max_, 2%; *D*_2%_, and mean; *D*_mean_), volumes of GTV and PTV (at 30 Gy; *D*_30Gy_, 33 Gy; *D*_33Gy_, and 36 Gy; *D*_36Gy_ [%]), conformity index,^31^, ^32^ gradient index,^32^ heterogeneity index,^33^ if simultaneously‐integrated boost (SIB) was used (Y vs N), if CTV was used (Y vs N), doses and volumes of OARs (for brain; *D*_max_, *D*_mean_, *D*_min_, *D*_2%_, *D*_98%_, *D*_10%_ [all in Gy], *V*_24Gy_ [cc], *V*_10Gy_ [%], for skin and palate mucosa; *D*_max_, *D*_mean_, *D*_2%_, *D*_98%_ [all in Gy], *V*_24Gy_ [cc], *V*_21Gy_ [cc], for optic chiasm/nerve; *D*_max_, *D*_mean_, *D*_2%_, *D*_98%_ [all in Gy], for left and right eyes [analyzed separately]; *D*_2%_, *D*_98%_ [all in Gy], *V*_10Gy_ [%]). All statistical analyses were performed by using commercially available software (SigmaPlot 13, Systat Software, Inc, San Jose, CA). *P*‐values <.05 were considered statistically significant. Overall survival time (OST) and disease‐specific ST (DSST) were assessed by Kaplan‐Meier analysis. Overall survival time was defined as days from the first day of SRT to death by any cause. Dogs that were alive or lost to follow‐up were censored. An analysis of DSST was performed by censoring tumor/treatment‐unrelated death in addition to those that were alive or lost to follow‐up. For evaluation of possible prognostic indicators, univariate Cox proportional hazard analysis was performed first. Multivariate Cox proportional hazard analysis was then performed by including variables with *P* < .05 in the univariate analysis.

## 3 RESULTS

Total 182 dogs met inclusion criteria. Among them, 175 dogs underwent 1 course of SRT and 7 dogs underwent 2 courses of SRT.

### 3.1 One hundred and seventy‐five dogs treated with 1 course of SRT

Among the 175 dogs that underwent 1 course of SRT, there were 88 neutered males, 5 intact males, 81 spayed females, and 1 intact female. Median age was 10 years (range: 2‐16 years old), and median body weight was 25.7 kg (range: 3‐68 kg). Tumor histopathology included 104 carcinomas (47 adenocarcinomas, 46 carcinomas, 10 squamous cell carcinomas, 1 transitional cell carcinoma), 53 sarcomas (23 chondrosarcomas, 14 osteosarcomas, 11 sarcomas with no further classification, 3 fibrosarcomas, 2 multilobular osteochondrosarcomas), and 18 other histology identifications (2 carcino‐sarcomas, and 1 plasma cell tumor and 15 with nondiagnostic samplings). The 15 dogs with nondiagnostic biopsy/cytology were included in the study because of CT characteristics were strongly suggestive of tumor, including boney lysis (the turbinates, other bones surrounding nasal cavity, or both), cribriform extension, mass effect, or combination of them, as evaluated by ACVR certified radiologists. Breeds included 48 mixed breed dogs, 19 Golden Retrievers, 17 Labrador Retrievers, 6 each of Bichon Frise, English Cocker Spaniel, 5 each of Australian Shepherd, and Miniature Schnauzer, 4 each of Border Collie, Boxer, German Shepherd, Shetland Sheep Dog, 3 Siberian Huskies, 2 each of Alaskan Malamute, Beagle, Cairn Terrier, Collie, Old English Sheep Dog, pug, Shih tzu, and Standard Poodle, and 1 Airedale Terrier, Australian Kelpie, Bearded Collie, Boston Terrier, Australian Cattle Dog, Chihuahua, Chow Chow, Dalmatian, English Bull Dog, English Springer Spaniel, Eskimo, German Wirehaired Pointer, Great Dane, Grey Hound, Great Pyrenees, Havanese, Italian Grey Hound, Lhasa Apso, Maltese, Miniature Dachshund, Miniature Poodle, Newfoundland, Pharaoh Hound, Pomeranian, Puli, Rottweiler, Samoyed, Scottish Terrier, Viszla, Welsh Corgi, Welsh Terrier, West Highland White Terrier, Norwegian Elkhound, and Yorkshire Terrier.

Among the 175 dogs, thoracic radiographs were performed as part of the initial staging in 162 dogs and no radiographic evidence of lung metastasis was detected. In 13 dogs, information regarding thoracic radiographs was not recorded. Aspirates of regional LNs revealed evidence of metastatic spread to lymph nodes in 8 dogs; 4 adenocarcinomas, 3 carcinomas, and 1 squamous cell carcinoma, and reactive LNs in 51 dogs. In 116 dogs, regional LNs were not aspirated or information was unavailable. Regional LN aspirates revealed evidence of regional spread in 8 dogs and 8 dogs were thought to have LN spread because of lymphadenopathy identified on imaging by contrast enhancement, enlargement, heterogeneity, or combination of them. Lymph nodes were included in the treatment field in these 16 dogs. LN were not irradiated in 159 dogs. Among the 16 dogs with LN involvement, PTV expansions for the LNs were isotropic 2 mm expansion in 10 dogs, and the last 6 dogs were contoured to the recommended expansions in a publication that evaluated LN movement during treatment of head and neck cancers (mandibular LNs: 0.7 cm right‐left, 0.5 cm dorso‐ventral, and 0.8 cm cranio‐caudal. retropharyngeal LNs: 0.6 cm right‐left, 0.4‐0.8 cm dorso‐ventral, and 0.5 cm cranio‐caudal.)^34^ Prescription to the nodes was the same as to the nasal tumor, however, that dose could not be met because of OAR constraints.^7^ Median and mean *D*_98%_ of the regional LNs in those 16 dogs were 21.5 and 22.1 Gy (range: 17.6‐29.9 Gy), respectively. Because of the small number of dogs with LN regional extension, this was not included in the prognostic indicator analysis.

Information about usage of steroids, nonsteroidal anti‐inflammatory drugs, and antibiotics and their duration were not consistently available because of the retrospective nature of this study. Therefore, those were not included in the further analyses.

Among the 175 dogs, 4 dogs were modified Adams stage 1, 5 dogs were stage 2, 81 dogs were Stage 3, and 85 dogs were stage 4 (4a = 44, 4b = 41). The volumes of target structures and calculated radiation doses/volumes to those structures are summarized in Table 1.

**TABLE 1:** Summary of dose and volume data in 175 dogs that received 3 fractions of 10 Gy SRT for sinonasal tumors.

		Median	Mean	SD	Minimum	Maximum
GTV	Volume (cc)	51.5	64.1	56.2	3.7	395
	*D*_max_ (Gy)	36.9	37.4	2.7	32.2	47.4
	*D*_mean_ (Gy)	32.5	33	1.4	30.7	38.5
	*D*_min_ (Gy)	23.4	2.6	4.5	8.9	30.2
	*D*_2%_ (Gy)	34.6	35.3	2.1	31.6	43.2
	*D*_98%_ (Gy)	30.1	29.4	2.2	18.1	33.1
	*V*_30Gy_ (%)	98.1	96.3	4.4	78.3	100
	*V*_33Gy_ (%)	36.2	42.4	30.8	0	98.2
	*V*_36Gy_ (%)	0.05	8.9	19	0	86.4
PTV	Volume (cc)	105.2	115.1	75.6	9.2	461
	*D*_max_ (Gy)	37.1	37.6	2.6	32.4	47.4
	*D*_mean_ (Gy)	31.7	31.9	1.1	29.5	36.1
	*D*_min_ (Gy)	14.6	14.5	4.6	1.2	25.1
	*D*_2%_ (Gy)	34.5	35.2	2.04	31.5	42.6
	*D*_98%_ (Gy)	25.7	25	2.8	13.6	30.1
	*V*_30Gy_ (%)	85.4	84	8	50.3	98.1
	*V*_33Gy_ (%)	25.7	30.7	23	0	80.3
	*V*_36Gy_ (%)	0.038	5.5	11.7	0	55.8
	Conformity index	0.8	0.78	0.09	0.1	0.93
	Gradient index	2.8	3.04	0.95	2.02	9.5
	Homogeneity index	6.9	7.6	3.1	3.5	17.6
Brain	*D*_max_ (Gy)	31.6	30.5	7.8	0.5	42.6
	*D*_mean_ (Gy)	4.8	5	2.9	0.06	15.3
	*D*_min_ (Gy)	0.2	1.2	7.7	0.002	4.07
	*D*_2%_ (Gy)	22	20.1	6.1	0.23	32.4
	*D*_98%_ (Gy)	0.3	0.9	2	0.01	15.3
	*V*_24Gy_ (cc)	0.88	1.03	1.4	0	16.4
	*V*_10Gy_ (%)	15.7	18.6	16	0	99.2
Skin	*D*_max_ (Gy)	30.1	30.1	1.8	22.4	36.5
	*D*_mean_ (Gy)	3.5	3.8	1.6	1.3	9
	*D*_2%_ (Gy)	20.7	20.3	2.7	9.5	27.1
	*D*_98%_ (Gy)	0.01	0.06	0.18	0	1.6
	*V*_24Gy_ (cc)	1.1	1.7	1.8	0	13.3
	*V*_21Gy_ (cc)	5.4	5.9	3.4	0.02	23.4
Palate mucosa	*D*_max_ (Gy)	27.1	27	2.6	14.7	35.9
	*D*_mean_ (Gy)	16.6	16.2	7.2	1.1	22.1
	*D*_2%_ (Gy)	24.1	24	3.5	11	29.7
	*D*_98%_ (Gy)	4.2	5.6	5.2	0	16.6
	*V*_24Gy_ (cc)	0.2	0.4	0.9	0	10
	*V*_21Gy_ (cc)	1.7	1.9	1.4	0	12.8
Optic chiasm	*D*_max_ (Gy)	17.2	16.1	6.9	0	35.4
	*D*_mean_ (Gy)	9.8	9.8	4.8	0	23.8
	*D*_2%_ (Gy)	15.6	14.9	6.6	0	34.8
	*D*_98%_ (Gy)	6.5	6.5	3.9	0	26.5
Eye (left)	*D*_2%_ (Gy)	11.6	12.3	3.8	0.9	28
	*D*_98%_ (Gy)	4.6	4.6	1.8	0	13.8
	*V*_10Gy_ (%)	8.1	13.8	17.7	0	100
Eye (right)	*D*_2%_ (Gy)	11.6	12.4	4.4	0.94	29.2
	*D*_98%_ (Gy)	4.8	4.7	1.9	0.2	14.4
	*V*_10Gy_ (%)	8.4	15.8	22	0	100

CTV expansion was used in 140 dogs and was not used in 35 dogs. SIB was used in 82 dogs and not used in 93 dogs. Typically, SIB was used to create an intentional hot spot (20%‐30% of prescription) inside the GTV.

In 174 dogs, isotropic 2 mm PTV expansion was used and in 1 dog, 1 and 2 mm isotropic PTV expansion was used for intracranial and extracranial components, respectively.

SRT plans consisted of 6 (N = 3), 7 (75), 8 (17), 9 (30), 10 (14), 11 (22), 12 (8), 13 (3), 15 (1), 17 (1), and 18 (1) photon beams. Beam energy used was 6 MV beams in 159 dogs and both 6 and 10 MV beams in 16 dogs. Coplanar beam arrangement was used in 139 dogs and noncoplanar in 36 dogs. Bolus was not used in any dogs. In each treatment session, dog position was verified via CBCT match in 136 dogs and orthogonal KV‐KV match in 39 dogs. The SRT course was completed in median 3 days (range: 3‐8 calendar days).

### 3.2 Treatment outcome

**FIGURE 2:**
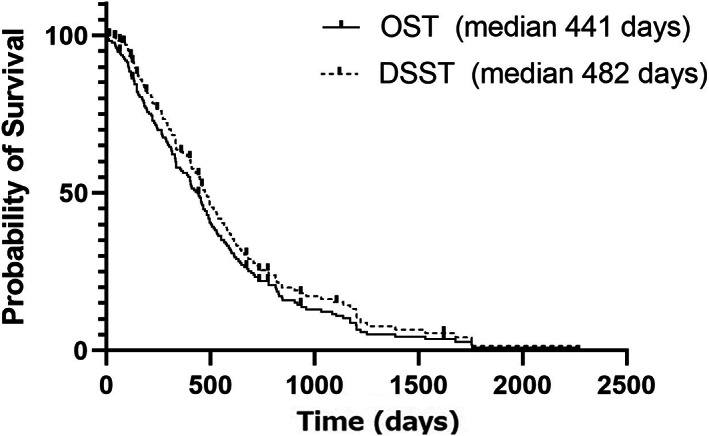
A Kaplan‐Meier graph showing overall survival time and disease‐specific survival time in 175 dogs with sinonasal tumor treated with 1 course of SRT (3 fractions of 10 Gy). Ticks indicate censored cases.

At the time of data analysis, 142 dogs of 175 dogs (81.1%) died. The median OST was 441 days (lower and upper 95% confidence interval [CI]; 389‐493 days) and 14 dogs were censored (alive = 3, lost to follow up = 11). Median follow up period of the 14 censored dogs was 498 days (range: 64‐1622 days). The median DSST was 482 days (95% CI: 428‐536 days; Figure 2) with 37 dogs (21.1%) censored with median follow up period of 314 days (range: 4‐1622 days). Among the 37 censored dogs, the reason of censoring included: alive/lost to follow up (14) and death because of obviously tumor/treatment‐unrelated reasons (23). Among those 23 dogs, reasons of euthanasia/death (and DSST) were hind limb weakness in 4 dogs, hepatitis/pancreatitis in 3 dogs, multicentric lymphoma in 2 dogs, pneumonia in 2 dogs, chronic renal failure in 2 dogs, and 1 each of chronic heart failure, hit by car, torn anterior cruciate ligament, ruptured lung bullae, gastric dilatation and volvulus, primary lung carcinoma, cutaneous plasma cell tumor, inflammatory bowel disease, heart failure, and severe arthritis. In the following analyses for a possible prognostic indicator, DSST was used to focus treatment and disease‐related outcomes.

Results of univariate Cox proportional hazard analysis are shown in Table 2. Based on values, age, BW, volume of GTV, PTV *D*_mean_, brain *D*_mean_, brain *D*_98%_, brain *D*_10%_, brain *V*_10Gy_, skin *D*_max_, left and right eye *D*_98%_, presence of clinical signs of nervous system after SRT (all N = 175) were included in the Cox multivariate proportional hazard analysis. Among them, brain *V*_10Gy_ (%) showed the strongest negative significance (*P* = .003) followed by presence of clinical signs of nervous system after SRT (*P* = .005), age, and brain *D*_mean_ (Gy; both *P* = .01; Table 3). Additionally, log rank analysis was then performed to calculate DSSTs of subgroups (ie, high vs low; divided by median value of the variable; Table 3). Chi‐square analysis to evaluate a correlation between presence of cribriform plate lysis and clinical signs of nervous system after SRT was not significant (*P* = 0.2). Blood work variables (Table S1) were not included in the multivariate analysis to avoid lowering statistical power because of smaller sample size.

**TABLE 2:** Summary of univariate Cox proportional hazard analyses in 175 dogs that received 3 fractions of 10 Gy SRT for sinonasal tumors.

			Cox univariate		
		N	Coefficient	Hazard ratio (95% CI)	*P*‐value
Sex	Intact male	5			.2
	Castrated male	88			
	Intact female	1			
	Spayed female	81			
Age		175	0.068	1.07 (1.00‐1.1)	**.05**
Body weight		175	0.02	1.02 (1.004‐1.04)	**.01**
Histology	Carcinomas	104			.95
	Sarcomas	55			
	Others	16			
Tumor laterality	Unilateral	64			.05
	Bilateral	111			
Orbital involvement	Yes	94			.71
	No	81			
Subcutaneous/submucosal involvement	Yes	94			.34
	No	81			
Nasopharyngeal involvement	Yes	122			.71
	No	53			
CP lysis	Yes	77			.62
	No	98			
Modified Adams stage	1	4			.5
	2	5			
	3	81			
	4	85			
	1/2/3	90			.64
	4	85			
	1/2/3/4a	134			.44
	4b	41			
CTV used?	With CTV	140			.53
	Without CTV	35			
LN treated?	Yes	16			.17
	No	159			
Tumor volume (cc)	GTV	175	0.0059	1.006 (1.003‐1.009)	**<.001**
	CTV	137	0.0051	1.005 (1.002‐1.009)	**.001**
	PTV	175	0.0045	1.005 (1.002‐1.007)	**<.001**
Tumor volume (cc)/body weight (kg)	GTV/BW	175	0.19	1.2 (1.08‐1.4)	**.001**
	CTV/BW	137	0.2	1.2 (1.07‐1.4)	**.004**
	PTV/BW	175	0.14	1.2 (1.05‐1.3)	**.002**
Conformity index		175			.99
Gradient index		175			.42
Homogeneity index		175			.98
SIB used?	With	82			.66
	Without	93			
GTV	*D*_max_ (Gy)	175			.18
	*D*_mean_ (Gy)				.11
	*D*_min_ (Gy)				.4
	*D*_2%_ (Gy)				.22
	*D*_98%_ (Gy)				.62
	*V*_30Gy_ (%)				.9
	*V*_33Gy_ (%)				.19
	*V*_36Gy_ (%)				.09
PTV	*D*_max_ (Gy)	175			.18
	*D*_mean_ (Gy)		0.16	1.2 (1.02‐1.4)	**.03**
	*D*_min_ (Gy)				.21
	*D*_2%_ (Gy)				.25
	*D*_98%_ (Gy)				.39
	*V*_30Gy_ (%)				.08
	*V*_33Gy_ (%)				.09
	*V*_36Gy_ (%)				.09
Brain	*D*_max_ (Gy)				.15
	*D*_mean_ (Gy)		0.097	1.1 (1.03‐1.2)	**.004**
	*D*_min_ (Gy)				.88
	*D*_2%_ (Gy)				.2
	*D*_98%_ (Gy)		0.092	1.1 (1.02‐1.2)	**.01**
	*D*_10%_ (Gy)		0.025	1.03 (1.007‐1.05)	**.007**
	*V*_24Gy_ (cc)				.98
	*V*_10Gy_ (%)		0.024	1.02 (1.01‐1.04)	**<.001**
Skin	*D*_max_ (Gy)		0.099	1.1 (1.007‐1.2)	**.04**
	*D*_mean_ (Gy)				.08
	*D*_2%_ (Gy)				.48
	*D*_98%_ (Gy)				.11
	*V*_24Gy_ (cc)				.64
	*V*_21Gy_ (cc)				.06
Palate mucosa	*D*_max_ (Gy)				.55
	*D*_mean_ (Gy)				.48
	*D*_2%_ (Gy)				.41
	*D*_98%_ (Gy)				.75
	*V*_24Gy_ (cc)				.62
	*V*_21Gy_ (cc)				.16
Optic chiasm	*D*_max_ (Gy)				.67
	*D*_mean_ (Gy)				.96
	*D*_2%_ (Gy)				.97
	*D*_98%_ (Gy)				.92
Left eye	*D*_2%_ (Gy)				.15
	*D*_98%_ (Gy)		0.1	1.1 (1.0‐1.2)	**.05**
	*V*_10Gy_ (%)				.18
Right eye	*D*_2%_ (Gy)				.28
	*D*_98%_ (Gy)		0.1	1.1 (1.0–1.2)	**.02**
	*V*_10Gy_ (%)				.32

*Note*: Bold indicates *P*<0.05.

**TABLE 3:** Summary of multivariate Cox proportional hazard analyses in 175 dogs that received 3 fractions of 10 Gy SRT for sinonasal tumors.

	Kaplan‐Meier (log rank)					Cox multivariate		
	Group	N	Median DSST (days)	95% CI	*P*‐value	Coefficient	Hazard ratio (95% CI)	*P*‐value
**Age**	Low	97	521	428‐614	0.047	0.1	1.1 (1.02‐1.18)	**.01**
	High	78	428	136‐308				
Body weight								.06
GTV volume (cc)								.96
PTV *D*_mean_ (Gy)								.08
**Brain** ***D***_**mean**_ **(Gy)**	Low	91	514	369‐659	0.014	−0.26	0.77 (0.62‐0.95)	**.01**
	High	84	449	377‐521				
Brain *D*_98%_ (Gy)								0.2
Brain *D*_10%_ (Gy)								.14
**Brain** ***V***_**10Gy**_ **(%)**	Low	87	592	456‐728	0.005	0.06	1.06 (1.02‐1.1)	**.003**
	High	88	441	376‐506				
Skin *D*_max_ (Gy)								.06
Eye left *D*_98%_ (Gy)								.79
Eye right *D*_98%_ (Gy)								.62
**Clinical signs of nervous system after SRT**	No	155	495	420‐569	0.002	Reference group		**.005**
	Yes	20	311	251‐371		0.8	2.2 (1.3‐4.0)	

*Note*: Bold indicates *P*<0.05.

Mann‐Whitney Rank Sum test was performed to evaluate if there was difference in dose/volume parameters of the brain between modified Adams stages (Table S2). When compared between stages 1‐3 and stage 4, stages 1‐3 had significantly lower dose/volume parameters in all analyses (*P* < .001) compared to stage 4. Same was true when compared between stages 1‐4a vs 4b (*P* < .001).

Mann‐Whitney Rank Sum test was also performed to evaluate if there was a significant difference in target dose and volume parameters between stage 1‐3 and 4, and between 1‐4a and 4b (Table S3). GTV *D*_min_, GTV *D*_98%_, GTV V_30Gy_, and PTV *D*_min_ were significantly lower in stage 4 (vs stage 1‐3) and stage 4b (vs stage 1‐4a), and PTV V_30Gy_ and PTV *D*_98%_ were significantly lower in stage 4 (vs stage 1‐3).

### 3.3 Seven dogs that underwent 2 courses of SRT

Median age and BW of the 7 dogs that underwent 2 courses of SRT were 10 years old (range: 8‐14 years old) and 32.4 kg (9.3‐43 kg), respectively (at the first SRT course). Breeds included 2 Golden Retrievers, 2 German Shepherds, and 1 American Eskimo, Siberian husky, and Pug. The group included 5 neutered males, 1 intact male and 1 spayed female. There were 3 adenocarcinomas, 1 each of carcinoma, osteosarcoma, chondrosarcoma, and sarcoma. At the time of first SRT course, there were 1 dog with modified Adams stage 2, 2 dogs with Stage 3, and 4 dogs with stage 4. At the time of the second course of SRT, 1 dog was Stage 3 (originally stage 2) and 6 dogs were stage 4. One dog received the same 3 daily fractions of 10 Gy in the second SRT course and 6 dogs were treated with an every other day schedule in hopes of ameliorating acute radiation adverse events.^35^

After the second course of SRT, 6 dogs were euthanized because of signs associated with the sinonasal tumor clinically determined to be progressive disease. The last dog was euthanized because of difficulty to stand, but without clinical signs associated with progressive disease. Median OST of those 7 dogs after the first SRT course (until euthanasia) was 498 days (95% CI: 464‐531 days). Median duration between the second SRT course and euthanasia was 139 days (95% CI: 49‐229 days). No dog was censored in this analysis. Median and mean durations between the first and second SRT courses were 401 days and 417 days (range: 286‐619 days), respectively. The mean duration between second course of SRT and death relative to the duration between first and second courses of SRT was 40% (range: 12.5%‐76%).

### 3.4 Radiation adverse events

Among the 175 dogs treated with a single course of SRT, information about acute adverse events was available in 132 dogs (75%, Table 4). Four dogs (3.0%) developed grade 2 or 3 skin or oral mucosa acute side effects. One dog developed temporal head tilt (Grade 1 CNS) 2 weeks after completion of SRT that improved within 2 weeks.

**TABLE 4:** Summary of acute radiation adverse events in 132 dogs that received 3 fractions of 10 Gy SRT for sinonasal tumors.

N = 132	Grade 0	Grade 1	Grade 2	Grade 3
Skin/hair	77 (58%)	51 (39%)	4 (3%)	0
Mucous membrane/oral cavity	118 (89%)	10 (8%)	3 (2%)	1 (1%)
Eye	115 (87%)	12 (9%)	4 (3%)	1 (1%)
Ear	132	0	0	0
CNS	131 (99%)	1 (1%)	0	0

Development of chronic rhinitis after SRT was recorded in 54 of 88 dogs (61%) for which such information was available. Rhinitis was defined as nasal muco+/−purulent discharge sometimes associated with sneezing, epistaxis, or both. Treatment recommendations include anti‐inflammatory drugs, and an antibiotic regimen. The severity and duration of chronic rhinitis was not consistently available because of the retrospective nature of the study. Three of the SRT treated dogs developed confirmed fungal rhinitis.

**FIGURE 3:**
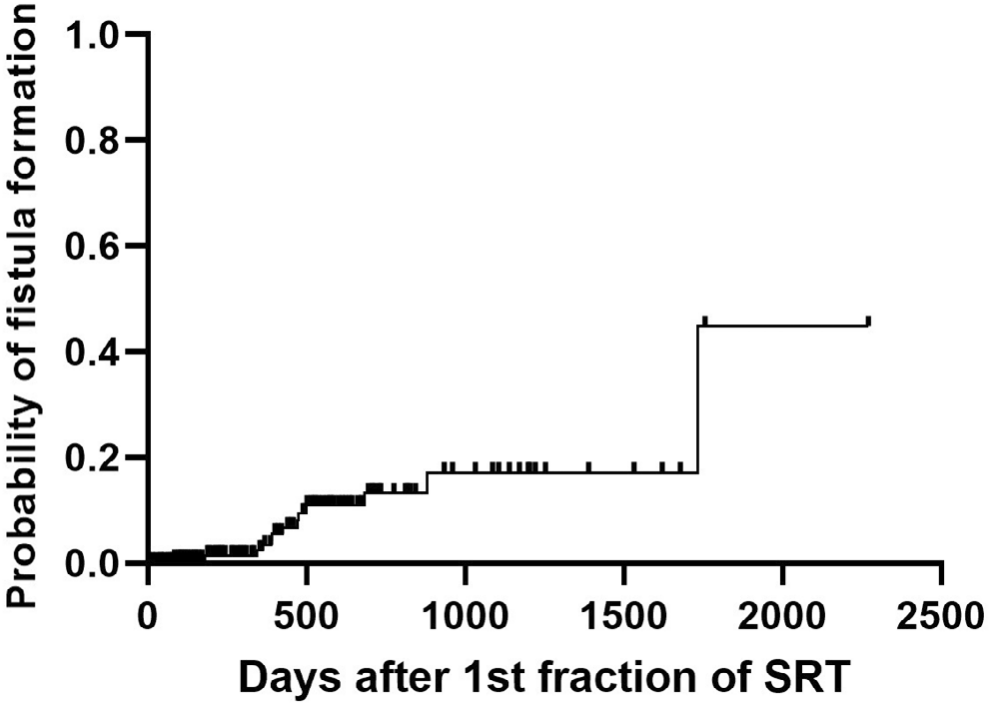
Cumulative risk curve for fistula formation after SRT. Ticks indicate censored cases.

Among the 182 dogs, oro‐nasal or naso‐cutaneous fistula development after SRT was noted in 13 dogs (7.1%). Those 13 dogs received only 1 course of SRT. Median and mean times to develop fistula after SRT were 425 days and 532 days, respectively (range: 83‐1733 days; Figure 3). Two of the fistulas were oro‐nasal and both dogs had severe palatial lysis before SRT. Of the remaining 11, 6 had oro‐subcutaneous involvement with lysis of the bone underlying the skin before SRT. None of the 13 dogs were censored in the survival analysis. Cox univariate analysis (including only the 175 dogs that underwent only 1 course of SRT) revealed no statistical significance in STs between 13 dogs (592 days) that developed fistula after SRT and 162 dogs (453 days) that did not (*P* = .41). Among the 13 dogs, CTV expansion was not used in 3 dogs (3/35 dogs without CTV expansion; 8.6%) whereas CTV expansion was used in 10 dogs (10/140 dogs with CTV expansion; 7.1%) and there was no significant difference in STs.

Among the 182 dogs, 20 dogs (11%) developed clinical signs of nervous system, primarily seizures (N = 18), after SRT (Table 5). Those 20 dogs were not censored in the OST and DSST analyses and all received only 1 course of SRT. Among those 20 dogs, 1 each dog was in modified Adams stage 1 and 2, 5 dogs in Stage 3 and 13 dogs were in stage 4 (4a = 6, 4b = 7). Among the 13 dogs with stage 4 disease, 1 dog (stage 4b) also had seizure activity as initial chief complaint along with sneezing and epistaxis. Wilcoxon Rank Sum analysis was used to investigate if the 20 dogs that developed clinical signs of nervous system after SRT had a significantly higher value in any of the brain variables compared to the 155 dogs that did not develop them (included only 175 dogs that underwent 1 course of SRT). Those 20 dogs had higher brain *D*_mean_ than the 155 dogs (5.8 Gy vs 4.7 Gy, respectively, *P* = .02), but lower GTV *D*_min_ (21.6 Gy vs 23.6 Gy, *P* = .04), GTV *D*_98%_ (29.4 Gy vs 30.2 Gy, *P* = .01), GTV *V*_30Gy_ (96.4 cc vs 98.4 cc, *P* = .009), and PTV *D*_min_ (11.3 Gy vs 14.8 Gy, *P* = .002) than the 155 dogs.

**TABLE 5:** Summary of 20 dogs that developed clinical signs of nervous system after SRT.

Case #	Reason of death/euthanasia	Overall survival time	Modified Adams stage	Note
1	No detail available	573	4b	Large intracranial invasion noted before SRT
16	Seizure probably because of PD	183	4b	Presented with seizure as part of the chief complaint
18	Seizures, vomit and anorexia, developed hemangiosarcoma on 3rd eyelid.	422	3	
28	Seizure	205	4a	
58	Seizure	302	3	Also had thyroid carcinoma, received carboplatin after SRT
60	Seizure	521	4a	
90	No detail available	491	2	No further diagnostics pursued
95	Seizure	465	4a	Also developed naso‐cutaneous fistula
100	Seizure	695	4a	No further diagnostics pursued
110	Seizure	616	3	No further diagnostics pursued
112	Seizure	153	1	First seizure noted 2 weeks after SRT
119	Seizure	311	4b	Large intracranial invasion noted before SRT
127	Seizure	52	4a	
128	Seizure	293	3	
136	Seizure	599	3	Also developed naso‐cutaneous fistula
146	Seizure	334	4a	No further diagnostics pursued
147	Seizure	182	4b	Large intracranial invasion noted before SRT
149	Seizure	146	4b	
162	Seizure	502	4b	Caudal brainstem tumor incidentally found and simultaneously irradiated (8 Gy × 3fx)
166	Seizure	173	4b	Large intracranial invasion noted before SRT

Abbreviation: PD, progressive disease.

In the 7 dogs that underwent 2 courses of SRT, information about acute adverse events after first SRT course was available in 6 dogs and information about them after second SRT course was available in 5 dogs, respectively. After the first and second SRT courses, 1 and 3 dog(s) developed VRTOG grade 1 acute skin adverse events, respectively. All acute adverse events did not require treatment.

## 4 DISCUSSION

This study of 182 dogs supports the hypothesis that SRT can be safely administered and is well tolerated. Acute adverse events were absent or minimal, even in dogs treated with a second course of therapy. OS and DSST were comparable to previous reports of treatment using fractionated IMRT.^5^, ^30^

Radiotherapy is used for decades for both palliative and definitive treatment for sinonasal tumors in dogs, including work before the invention of the CT and MV radiotherapy. Over time, veterinary radiotherapy transitioned to MV radiation and planning evolved from clinical set‐ups to 2D computer planning, and then 3D‐CRT planning. These advances did not improve severe acute radiation adverse events that were challenging for the dogs, the owners, and clinicians. The most important technical advancements for treatment of a tumor as complex in shape and size located in an anatomically intense region was IMRT and later volumetric arc modulated therapy (VMAT).^3^, ^13^, ^14^, ^18^, ^19^ Fractionated IMRT and VMAT decrease acute radiation adverse events and provide improved OS and DSST over earlier techniques. Stereotactic radiotherapy for nasal tumors still relies on IMRT and VMAT technologies but combines on‐board imaging and tumor and anatomic verification to decrease fraction number.

One of the most important aspects of this data was the improvement in the prognosis for dogs with cribriform lysis, extension of the tumor into the brain (Adams stage 4a and b), or both. Numerous studies over the years indicated that dogs with cribriform involvement at the time of treatment had a worse long‐term survival. For example, MV fractionated radiotherapy of dogs with cribriform plate involvement produces significantly shorter median ST of 6.7 months compared to 23.4 months in dogs with modified Adams stage 1.^29^ Later, a report of 29 dogs with stage 4 tumors treated with fractionated MV radiotherapyshowed improved OST of 318 days,^30^ which concurs with the Gieger 2017 publication with 29 dogs.^13^ However, a more recent paper by the same group with 129 dogs^24^ showed dogs with modified Adams stages 1‐4a had superior DSST than dogs with stage 4b. One possible reason for this difference between the Nolan study and the current study was our higher proportion of stage 4 tumors (85/175, 49%). This might be because of stage drift caused by improved CT imaging and more sophisticated image interpretation resulting in more tumors that would have been historically classified as Stage 3 being classified as stage 4a. We made the observation that while dogs with mild to modest cranial invasion did well, a subset of 5 dogs with extensive invasion did poorly among the stage 4b tumors. The modified Adams Staging criteria did not allow for exclusion of this subset and we were unable to quantify a specific volume or characteristic that led to poor outcome.

Twenty dogs developed clinical signs of nervous system near the time of death. When assessed based on the modified Adams stages before SRT, 1/4 dogs in stage 1 (25%), 1/6 dogs in stage 2 (17%), 5/81 dogs in Stage 3 (6%), and 6 dogs in stage 4a (6/24, 14%) and 7 in stage 4b (7/41, 17%) experienced clinical signs of nervous system after SRT. Nine out of those 20 dogs had OST longer than 12 months and the appearance of seizures or other signs of nervous system came close to time of euthanasia. Wilcoxon Rank Sum test revealed that dogs with those signs after SRT had statistically lower GTV *D*_min_, GTV *D*_98%_, GTV V_30Gy_, and PTV *D*_min_ than dogs without them after SRT. This finding might suggest that seizure activity observed in some of the dogs after SRT are because of failure to control tumor near the brain because of dose sparing near the brain. Conversely, increased PTV *D*_mean_, brain *D*_mean_, brain *D*_98%_, brain *D*_10%_, brain *V*_10Gy_, was associated with a worse prognosis. This provides 2 conflicting hypotheses for seizures near the end of life; tumor recurrence or brain necrosis.

Chronic rhinitis after treatment was seen in 61% of the cases in the current study where that information was available. The signs of the observed rhinitis were rarely clinically severe and typically manageable medically. These findings might be similar to those seen in fractionated IMRT radiotherapy, but lack of phase III studies prevents a comparison. Also, historically there is a lack of quantitative/qualitative reports of rhinitis.^14^, ^18^, ^19^ Three dogs (3/185) had fungal rhinitis confirmed after SRT and could be underrepresented because of lack of routine rhinoscopy.

Thirteen of 182 dogs (all 13 of which received only 1 course of SRT) developed fistulas. The median and mean time until appearance of the fistulas 425 days and 532 days, and all dogs had either evidence of severe palatal lysis (2) or extension into surrounding bones and subcutaneous tissues. These effects are not desirable and owners should be aware of risks when there is extensive palatial or bone/subcutaneous involvement.

In the 7 dogs that underwent 2 SRT courses, the duration between the 2nd SRT course and death was 40% in mean compared to the duration between the 1ST SRT and 2nd SRT courses. This finding is in accordance with our clinical impression; duration of local tumor control after the 2nd SRT course is shorter (40%) than that after the 1st SRT course. Ironically, no dogs treated with 2 courses of SRT developed fistulas, but based on Figure 3, time after treatment might be the most important factor driving fistula development. Alternate therapies, such as palliative radiation, chemotherapy or targeted therapy could offer comparable results.

The dogs treated in the early in the initiation of the protocol did not have a CTV expansion. SRT plans do not conventionally include a CTV. Two dogs with early recurrences were thought to have had geographic misses, instigating the addition of a CTV expansion. In retrospect our decision to include a CTV was made too early. This data showed no significant differences between the groups; however, the numbers in the non‐CTV group are not robust.

Several limitations exist in the current study, the most important of which is lack of follow‐up advanced imaging, necroscopy, or both. Precise information about clinical signs before and after SRT in many dogs was difficult to obtain, as was the use of adjuvant therapy such as chemotherapy, NSAIDs, and steroids. Collecting this information in a retrospective study can be challenging without a stringent follow‐up protocol. This study was also initiated during early experience with SRT for nasal tumors, leading to changes in the protocol over time.

In conclusion, SRT with 3 fractions of 10 Gy provides comparable tumor control to other SRT publication and fractionated IMRT publications and causes minimal acute adverse events. Survival times in dogs with stage 4a and 4b at the time of SRT were not significantly different than dogs with modified Adams stage 1‐3. Modified Adams stage alone might not be a reliable prognostic indicator for dogs with stage 4b. A modification seeking a more quantitative evaluation of tumor extending into the cranium should be pursued. CTV expansion might not be necessary.

## CONFLICT OF INTEREST DECLARATION

Authors declare no conflict of interest.

## OFF‐LABEL ANTIMICROBIAL DECLARATION

Authors declare no off‐label use of antimicrobials.

## INSTITUTIONAL ANIMAL CARE AND USE COMMITTEE (IACUC) OR OTHER APPROVAL DECLARATION

Authors declare no IACUC or other approval was needed.

## HUMAN ETHICS APPROVAL DECLARATION

Authors declare human ethics approval was not needed for this study.

## Supporting information


**Table S1.** Summary of blood works before SRT in 175 dogs that received 3 fractions of 10 Gy SRT for sinonasal tumors.

**Table S2.** Summary of Mann‐Whitney Rank Sum test to evaluate if there was difference in dose/volume variables of the brain between modified Adams stages.

**Table S3.** Summary of Mann‐Whitney Rank Sum test to evaluate if there was difference in dose/volume parameters of the GTV/PTV between modified Adams stages.
