# Supplementary material for: A retrospective study of sinonasal tumors in 182 dogs treated with stereotactic radiotherapy (3 × 10 Gy) (2010‐2015)
Source: J Vet Intern Med. 2023 Sep 8;37(6):2356–67. doi: 10.1111/jvim.16838 (PMC10658520; doi:10.1111/jvim.16838)
Supplement: Supplementary file 1 — Table S1. Summary of blood works before SRT in 175 dogs that received 3 fractions of 10 Gy SRT for sinonasal tumors. [file JVIM-37-2356-s001.pdf]

Table S1: Summary of blood works before SRT in 175 dogs that received 3 fractions of 10 Gy SRT for sinonasal tumors.

		N	Coefficient	Cox Univariate Hazard ratio (95% CI)	*P*-Value
	Nucleated cells	155			0.67
WBC ($x10^3$/ul)	bands	152			0.9
	segmented neutrophils	152			0.47
	lymphocytes	152			0.33
	monocytes	152			0.7
	eosinophils	152			0.4
	nRBC	151			0.32
RBC	HCT (%)	155			0.33
	RBC ($x10^6$/ul)	154			0.43
	cell HGB	152			0.06
	reticulocyte (x103/ul)	152	0.005	1.005 (1.001-1.009)	**0.02**
	PLT ($x10^3$/ul)	153			0.31
chemistry profile	Glu (mg/dl)	151			0.38
	BUN(mg/dl)	153			0.72
	Cre (mg/dl)	171			0.69
	Phos (mg/dl)	139	0.33	1.4 (1.1-1.8)	**0.014**
	Ca (mg/dl)	138			0.93
	Mg (mg/dl)	112			0.12

TP (g/dl)	155			0.61
Alb (g/dl)	151	-0.48	0.62 (0.41-0.93)	**0.022**
Glb (g/dl)	150	0.51	1.7 (1.1-2.4)	**0.008**
A/G ratio (nmol/L)	150	-0.85	0.43 (0.23-0.8)	0.007
Cholest (mg/dl)	134			0.91
T-bil (mg/dl)	141			0.53
ALP (IU/L)	149			0.7
ALT (IU/L)	150			0.93
AST (IU/L)	150			0.4
CK (IU/L)	120			0.14
GGT (IU/L)	148			0.21
Na (mEq/L)	139			0.59
K (mEq/L)	139			0.32
Cl (mEq/L)	133			0.91
HCO3- (mEq/L)	108			0.22
Anion gap (nmo/L)	111			0.081
Osmo (mOsm/kg)	112			0.46
