# Supplementary material for: A retrospective study of sinonasal tumors in 182 dogs treated with stereotactic radiotherapy (3 × 10 Gy) (2010‐2015)
Source: J Vet Intern Med. 2023 Sep 8;37(6):2356–67. doi: 10.1111/jvim.16838 (PMC10658520; doi:10.1111/jvim.16838)
Supplement: Supplementary file 2 — Table S2. Summary of Mann‐Whitney Rank Sum test to evaluate if there was difference in dose/volume variables of the brain between modified Adams stages. [file JVIM-37-2356-s003.pdf]

**Table S2:** Summary of Mann-Whitney Rank Sum test to evaluate if there was difference in dose/volume parameters of the brain between modified Adams stages.

| | Stage | Median | 25-75% | p-value |
|---|---|---|---|---|
| Brain $D_{max}$ (Gy) | 1-3 (N=90) | 30.9 | 28.7-32.3 | <0.001 |
| | 4 (N=85) | 32.2 | 31.1-33.5 | |
| | | | | |
| | 1-4a (N=134) | 31.4 | 29.2-32.5 | <0.001 |
| | 4b (N=41) | 32.6 | 31.3-33.4 | |
| | | | | |
| Brain $D_{mean}$ (Gy) | 1-3 (N=90) | 4.2 | 1.6-5.7 | <0.001 |
| | 4 (N=85) | 5.2 | 4.4-7.1 | |
| | | | | |
| | 1-4a (N=134) | 4.4 | 2.9-5.9 | <0.001 |
| | 4b (N=41) | 5.7 | 4.9-7.5 | |
| | | | | |
| Brain $D_{min}$ (Gy) | 1-3 (N=90) | 0.16 | 0.09-0.26 | <0.001 |
| | 4 (N=85) | 0.22 | 0.16-0.42 | |
| | | | | |
| | 1-4a (N=134) | 0.17 | 0.1-0.27 | <0.001 |
| | 4b (N=41) | 0.24 | 0.19-0.62 | |
| | | | | |
| Brain $D_{2\%}$ (Gy) | 1-3 (N=90) | 20.9 | 16.3-22.3 | <0.001 |
| | 4 (N=85) | 22.7 | 21.7-23.7 | |
| | | | | |
| | 1-4a (N=134) | 21.5 | 18.1-22.7 | <0.001 |
| | 4b (N=41) | 22.9 | 22.2-24.8 | |
| | | | | |
| Brain $D_{98\%}$ (Gy) | 1-3 (N=90) | 0.23 | 0.12-0.34 | <0.001 |
| | 4 (N=85) | 0.33 | 0.22-0.57 | |
| | | | | |
| | 1-4a (N=134) | 0.24 | 0.15-0.38 | <0.001 |
| | 4b (N=41) | 0.36 | 0.26-1.1 | |
| | | | | |
| Brain $D_{10\%}$ (Gy) | 1-3 (N=90) | 11.6 | 2.3-14.5 | <0.001 |
| | 4 (N=85) | 13.5 | 11.5-15.9 | |
| | | | | |
| | 1-4a (N=134) | 12.6 | 9.0-14.6 | <0.001 |
| | 4b (N=41) | 14.5 | 12.9-16.5 | |
| | | | | |
| Brain $V_{24Gy}$ (cc) | 1-3 (N=90) | 0.66 | 0.2-0.95 | <0.001 |
| | 4 (N=85) | 1.0 | 0.76-1.5 | |
| | | | | |
| | 1-4a (N=134) | 0.74 | 0.35-1.0 | <0.001 |
| | 4b (N=41) | 1.2 | 0.94-1.9 | |
| Brain $V_{10Gy}$ (%) | 1-3 (N=90) | 12 | 4.7-22.2 | <0.001 |

| | | | | |
|---|---|---|---|---|
| | 4 (N=85) | 17 | 12.4-28.4 | |
| | | | | |
| | 1-4a (N=134) | 13.3 | 7.8-21.9 | <0.001 |
| | 4b (N=41) | 18.3 | 15.1-36.4 | |
