# Supplementary material for: A retrospective study of sinonasal tumors in 182 dogs treated with stereotactic radiotherapy (3 × 10 Gy) (2010‐2015)
Source: J Vet Intern Med. 2023 Sep 8;37(6):2356–67. doi: 10.1111/jvim.16838 (PMC10658520; doi:10.1111/jvim.16838)
Supplement: Supplementary file 3 — Table S3. Summary of Mann‐Whitney Rank Sum test to evaluate if there was difference in dose/volume parameters of the GTV/PTV between modified Adams stages. [file JVIM-37-2356-s002.pdf]

**Table S3:** Summary of Mann-Whitney Rank Sum test to evaluate if there was difference in dose/volume parameters of the GTV/PTV between modified Adams stages.				
	Stage	Median	25-75%	p-value
GTV D_{max} (Gy)	1-3 (N=90)	36.8	35.4-38.8	0.15
	4 (N=85)	37	35.9-38.8	
	1-4a (N=134)	36.9	35.5-38.8	0.52
	4b (N=41)	36.9	36-38.3	
GTV D_{mean} (Gy)	1-3 (N=90)	32.6	32.1-33.7	0.75
	4 (N=85)	32.5	32-33.6	
	1-4a (N=134)	32.6	32.1-33.6	0.35
	4b (N=41)	32.4	32-33.6	
GTV D_{min} (Gy)	1-3 (N=90)	25.1	22.2-27.2	**<0.001**
	4 (N=85)	21.5	18.5-24.7	
	1-4a (N=134)	24.2	20.6-26.4	**<0.001**
	4b (N=41)	20.6	17.3-24.7	
GTV $D_{2\%}$ (Gy)	1-3 (N=90)	34.6	33.7-36.3	0.18
	4 (N=85)	34.8	34.1-36.5	
	1-4a (N=134)	34.6	33.8-36.5	0.6
	4b (N=41)	34.9	34.1-35.9	
GTV $D_{98\%}$ (Gy)	1-3 (N=90)	30.3	29.4-30.9	**0.007**
	4 (N=85)	29.7	28-30.5	
	1-4a (N=134)	30.2	29.4-30.8	**0.016**
	4b (N=41)	29.3	27.6-30.5	
GTV V_{30Gy} (%)	1-3 (N=90)	98.6	96.3-99.6	**<0.001**
	4 (N=85)	97.2	92.2-98.8	
	1-4a (N=134)	98.4	96.1-99.4	**0.004**
	4b (N=41)	96.6	89.8-99.1	
GTV V_{33Gy} (%)	1-3 (N=90)	37	12.5-72.1	0.87
	4 (N=85)	36.2	15.7-69.1	
	1-4a (N=134)	37.3	12.9-70.1	0.85
	4b (N=41)	33.8	15.1-69.6	

GTV V_{36Gy} (%)	1-3 (N=90)	0.013	0-3.9	0.3
	4 (N=85)	0.077	0-8.5	
	1-4a (N=134)	0.038	0-5.3	0.97
	4b (N=41)	0.062	0-1.1	
PTV D_{max} (Gy)	1-3 (N=90)	37	35.5-38.8	0.22
	4 (N=85)	37.1	36-38.9	
	1-4a (N=134)	37.1	35.7-38.8	0.63
	4b (N=41)	37	36-38.8	
PTV D_{mean} (Gy)	1-3 (N=90)	31.8	31.11-32.6	0.5
	4 (N=85)	31.7	31.1-32.6	
	1-4a (N=134)	31.8	31.1-32.6	0.403
	4b (N=41)	31.4	31.1-32.5	
PTV D_{min} (Gy)	1-3 (N=90)	15.6	12.8-18.5	**<0.001**
	4 (N=85)	13	10.1-16.2	
	1-4a (N=134)	15.1	11.3-17.9	**0.035**
	4b (N=41)	13.7	10.5-15.3	
PTV $D_{2\%}$ (Gy)	1-3 (N=90)	34.4	33.6-36.1	0.23
	4 (N=85)	34.6	33.9-36.5	
	1-4a (N=134)	34.5	33.7-36.2	0.66
	4b (N=41)	34.8	33.9-35.8	
PTV $D_{98\%}$ (Gy)	1-3 (N=90)	26.3	23.9-27.6	**<0.001**
	4 (N=85)	24.9	22.7-26.2	
	1-4a (N=134)	25.8	23.6-27.3	0.056
	4b (N=41)	25.2	23.1-26.3	
PTV V_{30Gy} (%)	1-3 (N=90)	86.4	81.9-91.4	**0.001**
	4 (N=85)	83.1	77.5-88.7	
	1-4a (N=134)	85.9	80.5-90.5	0.061
	4b (N=41)	82.7	77.5-89.5	
PTV V_{33Gy} (%)	1-3 (N=90)	24.5	9.3-47.1	0.71
	4 (N=85)	26.3	11.7-50.0	

	1-4a (N=134)	24.5	10-47.3	0.83
	4b (N=41)	26.3	11.1-53.9	
PTV V_{36Gy} (%)	1-3 (N=90)	0.015	0-2.03	0.33
	4 (N=85)	0.072	0-5.7	
	1-4a (N=134)	0.03	0-2.9	0.93
	4b (N=41)	0.051	0-0.87	
